# 

**Published:** 1861-11

**Authors:** 


### THE REGISTER.

We have on hand a complete set of the back volumes of the Dental Register, neatly bound, which we will sell for $3 a volume. This is the only set we are aware of that is obtainable. With us, except this, several of the earlier volumes are exhausted. For these, or subscription to the Register, we will take old gold, or platinum scraps, as readily as cash. For the latter we will pay $1.80 an ounce, and for gold according to the quality. J. TAFT.

### PATRIOTIC.

Our neighbor and professional brother, Dr. T. F. Davenport, has through a desire to serve his country in troublous times,

Laid down the Plugger and the Forceps
And taken up the gun and the sword.
(*That's blank verse.*)

We are sorry to lose the Dr. from our ranks, but presume he felt inclined to the performance of the "larger duty;" and we have the fullest confidence that his going forth will not be in vain.

May a shield of protection be over and around him. T.

### PERSONAL.

Dr. M. Wells, of Indiana, recent Demonstrator in the Ohio College of Dental Surgery, has returned to this city, and opened an office, intending to make this the field of his future operations. We are much pleased that he has become one of *us.* He is one of our *growing* dentists: he will not go to seed in a year or two. Success attend him.

### GIBSON HOUSE.

We take pleasure in directing attention to the Gibson House, because it abounds in all the elements of comfort and pleasure, to those who choose to become its inmates. Its arrangements in every particular are unexceptionable. We are pleased to see that it has become a kind of headquarters for the members of the dental profession visiting the city, and we can assure all that they will find no more pleasant and comfortable stopping place in the city, especially while it is under the auspices of our noble and patriotic citizen, Col. Geoffroy. This is the House at which if one stops, he will be quite sure to stop again. T.

### CONTENTS.

### DENTAL REGISTER,

### OF THE WEST.

### Issued Monthly, at $3 a year in advance.

DEVOTED TO THE INTERESTS OF THE DENTAL PROFESSION.

**Edited by J. TAFT and GEO. WATT.**

The Volume commences in January and closes in December.

The Register being issued monthly is always fresh, therefore indispensable to the practitioner who desires to keep posted up with the new inventions and improvements which are daily developed. Neither expense nor effort will be spared to give value and variety to its contents. Articles will be illustrated with well executed engravings, whenever they will add to the interest or assist in explanation.

Its extensive circulation and frequency of issue makes it the BEST DENTAL ADVERTISING MEDIUM in the United States.

☞ CONTRIBUTIONS SOLICITED.

Address Original Communications to **Geo. Watt, Xenia, O.**

Exchanges to **J. Taft**, or **Dental Register, Cincinnati, O.**

Business Communications, to

**JNO. T. TOLAND, Publisher and Proprietor,**
38 West Fourth Street, Cincinnati, O.

Communications must reach the Editor as early as the 15th, to insure insertion in the next issue.

